# Between adaptive and innate immunity: TLR4-mediated perforin production by CD28^null^ T-helper cells in ankylosing spondylitis

**DOI:** 10.1186/ar1840

**Published:** 2005-10-18

**Authors:** Bernd Raffeiner, Christian Dejaco, Christina Duftner, Werner Kullich, Christian Goldberger, Sandra C Vega, Michael Keller, Beatrix Grubeck-Loebenstein, Michael Schirmer

**Affiliations:** 1 Department of Internal Medicine, Innsbruck Medical University, Austria; 2 Ludwig Boltzmann Institute for Rehabilitation of Internal Diseases, Saalfelden, Austria; 3 Institute for Biomedical Aging Research, Austrian Academy of Science, Austria

## Abstract

CD3^+^CD4^+^CD28^null ^and CD3^+^CD8^+^CD28^null ^T cells are enriched in patients with immune-mediated diseases compared with healthy controls. This study shows that CD4^+^CD28^null ^T cells express Toll-like receptors recognizing bacterial lipopolysaccharides in ankylosing spondylitis, psoriatic arthritis and rheumatoid arthritis. In ankylosing spondylitis, TLR4 (23.1 ± 21.9%) and, to a smaller extent, TLR2 (4.1 ± 5.8%) were expressed on CD4^+^CD28^null ^T cells, whereas expression was negligible on CD4^+^CD28^+ ^and CD8^+ ^T cells. CD4^+^CD28^null ^T cells produced perforin upon stimulation with lipopolysaccharide, and this effect was enhanced by autologous serum or recombinant soluble CD14. Perforin production could be prevented with blocking antibodies directed against CD14 or TLR4. Incubation of peripheral blood mononuclear cells with tumour necrosis factor alpha led to an upregulation of TLR4 and TLR2 on CD4^+^CD28^null ^T cells *in vitro*, and treatment of patients with antibodies specifically directed against tumour necrosis factor alpha resulted in decreased expression of TLR4 and TLR2 on CD4^+^CD28^null ^T cells *in vivo*. We describe here a new pathway for direct activation of cytotoxic CD4^+ ^T cells by components of infectious pathogens. This finding supports the hypothesis that CD4^+^CD28^null ^T cells represent an immunological link between the innate immune system and the adaptive immune system.

## Introduction

Pattern recognition receptors (PRRs) are a family of receptors of the innate immune system binding to conserved pathogen-associated molecular patterns [1]. The most important PRRs are the Toll-like receptors (TLRs), which allow monocytes, neutrophils, dendritic cells, natural killer (NK) cells and B cells to recognize bacterial components, viruses, fungi and host material such as heat shock proteins [2-5]. Receptor engagement leads to the translocation of NF-κB and to gene transcription of proinflammatory cytokines. Lipopolysaccharide (LPS) from Gram-negative bacteria is the main ligand for TLR4. LPS binding to TLR4 is promoted by CD14, which can be present either in a membrane-bound form (mCD14) or a soluble form (sCD14) [6,7]. Whether LPS is a low-affinity ligand for TLR2 is still controversial [8].

The interactions between the innate and adaptive immune systems are crucial to promote proinflammatory reactions against pathogens and to ensure maintenance of vital self-tolerance. TLRs are expressed on both innate and adaptive immune cells and are critically involved in this interplay. TLR-stimulated dendritic cells induce specific T cells to differentiate into memory cells [9,10], and microbial induction of the TLR pathway on dendritic cells also blocks the suppressive effects of regulatory T cells [11,12]. In addition, TLRs are themselves expressed on T cells. In murine T cells, LPS signalling induces production of INF-γ and T-helper-1 accentuated immune responses [13,14]. After *in vitro *activation, mouse CD4^+ ^T cells express TLR3 and TLR9, and treatment of these cells with synthetic ligands for TLR3 and TLR9, viral dsRNA and bacterial unmethylated DNA enhances their survival [15]. LPS can directly activate regulatory T cells, and can thereby increase their suppressive function [16]. Activated human T cells express high levels of cell-surface TLR2 and produce elevated levels of cytokines in response to bacterial lipopeptide, a TLR2 ligand [17].

TLRs have also been shown to be important for the pathogenesis of immune-mediated diseases. In rheumatoid arthritis (RA), for example, TLR2 expression occurs in inflamed synovial tissue predominantly at sites of attachment and invasion into the cartilage and bone [18]. The TLR2-mediated stimulation of synovial fibroblasts with bacterial components promotes the release of proinflammatory cytokines and leads to a higher expression of TLR2.

In ankylosing spondylitis (AS), as in other immune-mediated diseases, an unusual proinflammatory and cytotoxic CD4^+ ^T-cell subgroup has been described, which lacks the co-stimulatory molecule CD28. These CD4^+^CD28^null ^T cells depend on alternative pathways for coactivation and can indeed obtain such signals by NK receptors recognizing ubiquitous major histocompatibility complex class I molecules [19-23]. Anomalous expression of NKG2D on these T cells together with upregulated MIC ligands in the inflamed synovial tissue of RA have also been shown to provide co-stimulatory signals [24]. CD4^+^CD28^null ^T cells have therefore been considered an immunological link between the adaptive and the innate defence system [25].

As CD4^+^CD28^null ^T cells have NK cell features, and as NK cells express TLRs on their surface [4], we investigated the expression of TLRs as an alternative stimulatory pathway on CD4^+^CD28^null ^T cells.

## Patients and methods

A total of 90 consecutive patients with spondyloarthropathies, 72 patients with RA and 64 age-matched healthy controls were enrolled into the study. Spondyloarthropathy was defined according to the European Spondyloarthropathy Study Group criteria [26]. Out of the spondyloarthropathy-defined patients, 65 patients had a diagnosis of AS according to the modified New York criteria [27] and 25 patients had psoriatic arthritis (PsA) as defined by the diagnostic criteria of Moll and Wright [28]. RA was diagnosed according to the criteria of the American College of Rheumatology [29]. Probands did not show any history, clinical or laboratory sign for infections nor malignant diseases. Healthy controls had no history of an immune-mediated disease. Heparinized blood samples were drawn from peripheral veins after informed and written consent according to the local ethics committee.

Patients' characteristics (including age, sex, the presence of rheumatoid factor and anti-cyclic citrullinated peptide antibodies, HLA-B27 status, axial involvement, erythrocyte sedimentation rate and C-reactive protein) are summarized in Table 1. Nine of the AS patients were treated with antibodies directed against tumour necrosis factor alpha (TNF-α) (infliximab = Remicade^®^; Aesca, Vienna, Austria) at a dosage of 3 mg/kg body weight.

### Cell preparation and surface staining

Peripheral blood mononuclear cells (PBMCs) were isolated by Ficoll density gradient centrifugation. For surface staining, PBMCs were incubated, as appropriate, with fluorescein isothiocyanate-conjugated anti-CD4, anti-CD8 or anti-CD28, with phycoerythrin-conjugated anti-CD28 and with peridinin chlorophyll protein-conjugated anti-CD3, anti-CD4 and anti-CD8 monoclonal antibodies (Becton Dickinson, San Diego, CA, USA). Specific mAbs directed against CD14 (fluorescein isothiocyanate), TLR4 and TLR2 (phycoerythrin; eBioscience, San Diego, CA, USA) were used to analyse expression of PRRs. Cells were incubated for 30 minutes at 4°C with the antibodies. After washing with PBS, cells were fixed in 4% paraformaldehyde (cellfix; Becton Dickinson). Stained cells were analysed on a FACS-Calibur analyser (Becton Dickinson). At least 100,000 events were counted for each acquisition. Data were analysed using WinMDI software (version 2.5, Joseph Trotter; Scripps Research Institute, La Jolla, CA, USA). Cells were considered positively stained when fluorescence levels were higher than those of the corresponding isotype controls.

For the isolation of CD4^+^CD28^+ ^and CD4^+^CD28^null ^T cells, the MACS^® ^CD4^+ ^T-cell multisort kit and magnetic bead labelled anti-phycoerythrin antibodies were applied according to the manufacturer's instructions (Miltenyi Biotech, Amsterdam, The Netherlands). To further increase the purity of T cells, sorted fractions were incubated in 24-well plates for 2 hours at 37°C to allow adherence of contaminating monocytes. Purity of isolated fractions was determined by flow cytometry as already described.

### Reverse transcription-polymerase chain reaction

Total RNA was extracted from isolated cell fractions using 1 ml Tri-Reagent (Sigma-Aldrich, St Louis, MO, USA), 200 μl chloroform (Sigma-Aldrich), 0.5 ml isopropanol (Sigma-Aldrich) and 1 μl glycogen (Roche, Basel, Switzerland) as previously described [30]. RNA was reverse transcribed to cDNA applying the Reverse Transcription System (Promega, Madison, WI, USA). PCR amplification was performed on 1 μg total RNA and 10 pmol primers (TLR2: forward, 5'-GGCCAGCAAATTACCTGTGTG-3'; reverse, 5'-CTGAGCCTCGTCCATGGGCCACTCC-3'; TLR4: forward, 5'-TGCAATGGATCAAGGACCAGAGGC-3'; reverse, 5'-GTGCTGGGACACCACAACAATCACC-3'; and β_2_-microglobulin: forward, 5'-CTCCGTGGCCTTAGCTGTG-3'; reverse, 5'-TTTGGAGTACGCTGGATAGCC-3') using the HotStar Master Mix Kit (Qiagen Valencia, CA, USA). The PCR reaction was performed according to a modified protocol [31] on the PTC-100 Thermal Cycler (MJ Research, Waltham, MA, USA) at 95°C for 3 minutes, at 95°C for 1 minute, 58°C for 2 minute and 72°C for 1 minute (35 cycles), and at 72°C for 2 minutes. Negative control samples were prepared by amplification reactions in the absence of cDNA. PCR products were separated on 1% agarose gel containing ethidium bromide and were visualized by UV illumination.

### LPS studies and intracellular cytokine staining

Isolated PBMCs were resuspended in RPMI 1640 containing 2 mM L-glutamine and were distributed on 24-well plates at a density of 1 × 10^6 ^cells per well. LPS of *Escherichia coli *serotype 026:B6 (Sigma) was added at a concentration of 10 μg/ml [16,17], sCD14 (R&D systems, Minneapolis, MN, USA) at 25 μg/ml and autologous serum at a concentration of 5% as indicated.

To test inhibitory effects of blocking anti-CD14 or anti-TLR4 antibodies, cells were resuspended in RPMI 1640 with 5% autologous serum and were incubated with 10 μg/ml blocking anti-CD14 antibody (R&D Systems), 10 μg/ml anti-TLR4 antibody (Torrey Pines Biolabs, TX, USA) and 10 μg/ml isotype control antibody (Becton Dickinson) for 1 hour before adding LPS at a concentration of 10 μg/ml. Brefeldin A (Sigma) was added to functional experiments at a concentration of 10 μg/ml to avoid release of produced cytokines. After incubation for 16 hours at 37°C the cells were washed, stained with CD4-peridinin chlorophyll protein mAbs and CD28-phycoerythrin mAbs and were permeabilized with 0.05% Tween 20 to stain intracellularly with fluorescein isothiocyanate-conjugated anti-perforin mAbs or control immunoglobulin (Becton Dickinson).

### Stimulation of short-term cell lines

Short-term cell lines were established after incubation of fresh PBMCs on stimulating immobilized anti-CD3 mAbs (OKT3; eBioscience) for 18 hours. Cells were then cultured in densities of 0.5 × 10^6^ – 2 × 10^6 ^in RPMI 1640 containing 10% FCS, 2 mM L-glutamine and 20 U/ml recombinant human IL-2 (Sigma). The medium was changed every 2–3 days. Cell lines were used after 7 days for stimulation assays over 24 hours with 20 ng/ml recombinant human TNF-α (Prepotech, London, UK).

### Enzyme-linked immunosorbent assay

Soluble levels of CD14 in blinded sera were tested in duplicates with an ELISA kit (R&D systems) according to the manufacturer's instructions.

### Statistical analysis

Statistical analysis was performed using the SPSS program (version 11.5; SPSS Inc., Chicago, IL, USA). The Kolmogorov–Smirnov test was used to test for normal distribution, and the Mann–Whitney *U* test and the Wilcoxon test were used as appropriate. At least six assays were performed for each experiment. *P *< 0.05 was considered significant and *P *< 0.01 was highly significant. Data are shown as box plots with the lines within the boxes representing the median, the boxes representing the 25th–75th percentiles and the lines outside the boxes including all values except mavericks.

## Results

### Comparison between the prevalences of circulating CD4^+^CD28^null ^and CD8^+^CD28^null ^T cells in AS, PsA, RA and healthy controls

**Figure 1:**
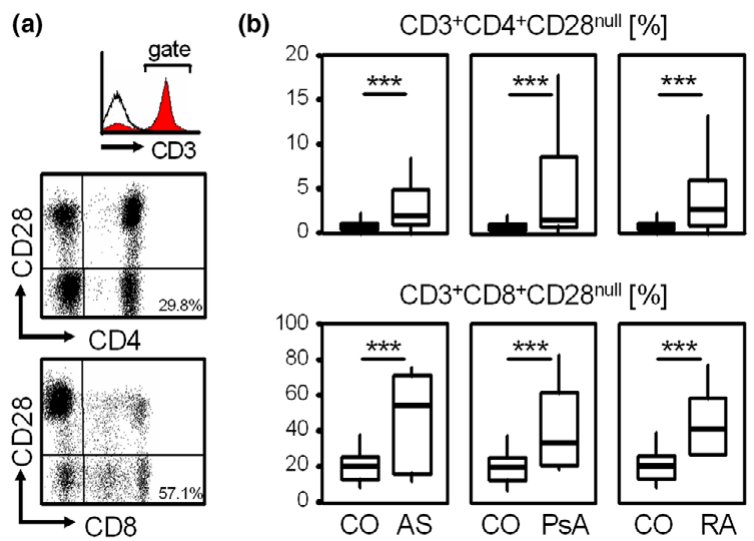
Prevalence of circulating CD3^+^CD4^+^CD28^null^**** and CD3^+^CD8^+^CD28^null^**** cells in ankylosing spondylitis, psoriatic arthritis and rheumatoid arthritis. **(a) **Representative example showing the prevalence of CD3^+^CD4^+^CD28^null ^T cells (percentage out of C3^+^CD4^+^) and CD3^+^CD8^+^CD28^null ^T cells (percentage out of C3^+^CD8^+^). The histogram shows staining for CD3 (filled grey curve) and isotype control antibody (black line). Dot plots are then gated on CD3^+ ^cells. **(b) **Box plots summarize the prevalence of CD3^+^CD4^+^CD28^null ^and CD3^+^CD8^+^CD28^null ^T cells in ankylosing spondylitis (AS), psoriatic arthritis (PsA) and rheumatoid arthritis (RA) patients. The Mann-Whitney *U* test was used to determine the statistical differences between patients and the age-matched healthy control group (CO). ***P < 0.001.

Peripheral blood from patients with AS, PsA and RA showed an increased prevalence of CD3^+^CD4^+^CD28^null ^T cells and CD3^+^CD8^+^CD28^null ^T cells compared with age-matched healthy controls. One representative example of a three-colour flow cytometry analysis showing the prevalence of CD3^+^CD4^+^CD28^null ^and CD3^+^CD8^+^CD28^null ^T cells is shown in Figure 1a. The mean percentages of CD3^+^CD4^+^CD28^null ^T cells out of CD3^+^CD4^+ ^T cells were 5.1 ± 9.8%, 5.1 ± 6.8%, 4.6 ± 5.2% and 1.5 ± 4.5% for AS, PsA, RA and healthy controls (each with *P *< 0.001), respectively. The mean percentages of CD3^+^CD8^+^CD28^null ^T cells out of CD3^+^CD8^+ ^T cells were 38.4 ± 21.9% in AS, 44.4 ± 21.7% in PsA and 46 ± 26.2% in RA, compared with 22.3 ± 12.4% in the control group (each with *P *< 0.001, Figure 1b).

### Increased expression of pattern recognition receptors on CD4^+^CD28^null ^T cells

**Figure 2:**
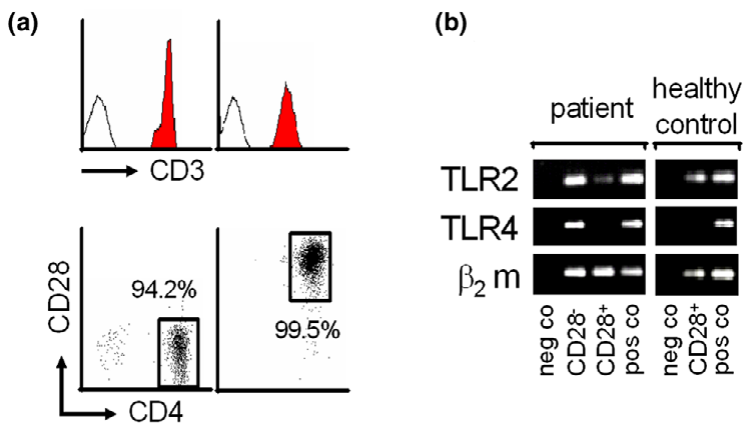
Messenger RNA expression of TLR2 and TLR4 in CD3^+^CD4^+^CD28^null ^and CD3^+^CD4^+^CD28^+ ^T cells. **(a) **Fluorescence-activated cell sorting analysis shows the purity of CD3^+^CD4^+^CD28^null ^and CD3^+^CD4^+^CD28^+ ^T cells. **(b) **mRNA expression of TLR2, TLR4 and β2-microglobulin (β2m, housekeeping gene) in CD3^+^CD4^+^CD28^null ^T cells (CD28^-^) and in CD3^+^CD4^+^CD28^+ ^T cells (CD28^+^). Peripheral blood mononuclear cells were used as positive control (pos co), and a negative control (neg co) was performed in the absence of cDNA. A representative example out of three independent experiments is given.

To test T cells for the expression of TLR2 and TLR4 mRNA, monocyte-depleted CD4^+^CD28^null ^and CD4^+^CD28^+ ^T cells were isolated from patients as well as from healthy controls (CD4^+^CD28^+ ^T cells). As shown in Figure 2a, purity was high for both with the CD3^+^CD4^+^CD28^null ^and CD3^+^CD4^+^CD28^+ ^fractions ranging between 94.2% and 99.7%. A representative example out of three independent RT-PCR experiments is depicted in Figure 2b: CD4^+^CD28^null ^T cells express TLR2 and TLR4 mRNA, whereas TLR4 transcripts were not detected in CD4^+^CD28^+ ^T cells from patients and healthy controls. In contrast, variable but significant levels of TLR2 mRNA were present in the CD3^+^CD4^+^CD28^+ ^population even from healthy individuals.

**Figure 3:**
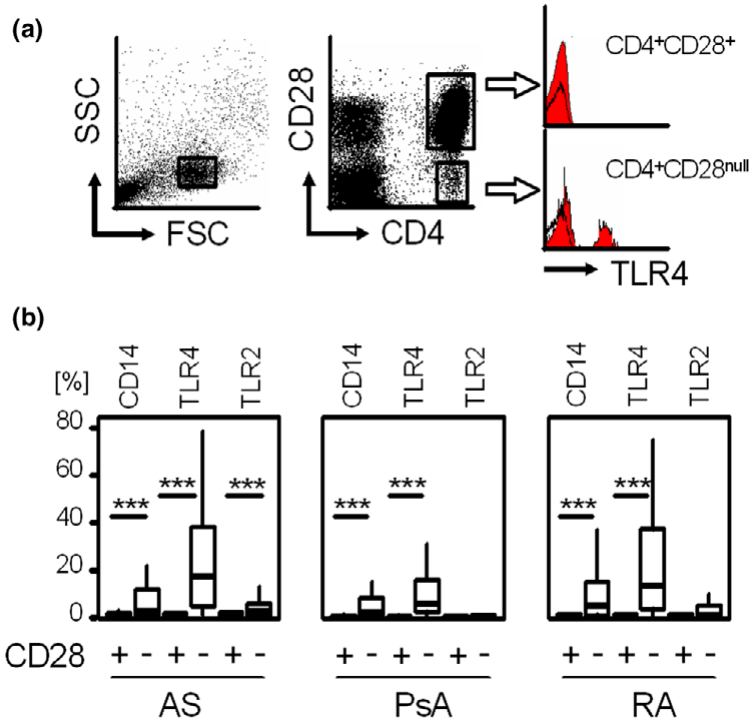
Surface expression of CD14, TLR4 and TLR2 on CD4^+^CD28^+ ^and CD28^null ^cells in ankylosing spondylitis, psoriatic arthritis and rheumatoid arthritis. **(a) **Representative dot plots and histograms show TLR4 expression (filled red curve, black line represents isotype control) on CD4^+^CD28^+ ^and CD4^+^CD28^null ^T cells. Gates were set on lymphocytes (forward scatter and sideward scatter) as well as on CD28^+ ^and CD28^null ^cells expressing high levels of CD4. **(b) **Box plots summarize the expression of CD14, TLR4 and TLR2 on CD4^+^CD28^+ ^and CD28^null ^T cells in patients as indicated. The Wilcoxon test was used to determine the statistical differences between the groups. ***P < 0.001. SSC, side scatter; FSC, forward scatter; AS, ankylosing spondylitis; PsA, psoriatic arthritis; RA, rheumatoid arthritis.

To address PRR surface expression on T cells, PBMCs from patients as well as from healthy controls were incubated with mAbs against CD4, CD14, CD28, TLR4 and TLR2, and were analysed by flow cytometry. As shown in Figure 3a, lymphocytes were gated on the forward and side scatter and specific gates were set to focus CD4^+^CD28^null ^and CD4^+^CD28^+ ^T cells for the analysis of TLR expression.

Overall, all analysed PRRs (CD14, TLR4 and TLR2) were detected on the surface of CD4^+^CD28^null ^T cells but not on CD28^+ ^T cells irrespective of the underlying diseases tested. CD14 was expressed on 13.3 ± 20.4% of CD4^+^CD28^null ^T cells versus 0.7 ± 1% of CD4^+^CD28^+ ^T cells in AS, on 8.8 ± 15.7% of CD4^+^CD28^null ^T cells versus 1.0 ± 2.3% of CD4^+^CD28^+ ^T cells in PsA, and on 11.3 ± 17.2% of CD4^+^CD28^null ^T cells versus 0.6 ± 0.6% of CD4^+^CD28^+ ^T cells in RA (each with *P *< 0.001). TLR-4, the main receptor for LPS recognition, was significantly expressed on CD4^+^CD28^null ^T cells in AS (23.1 ± 21.9% versus 0.9 ± 1.2%), in PsA (12.4 ± 18.1% versus 0.4 ± 0.5%) and in RA (23.1 ± 24.7% versus 0.6 ± 0.9%; each with *P *< 0.001). TLR2 was more frequently expressed on CD4^+^CD28^null ^T cells than on CD28^+ ^cells (4.1 ± 5.8% versus 1.0 ± 1.1%, *P *< 0.001) in AS, but not in PsA or in RA (Figure 3b). Negligible surface expression of PRRs without any difference between CD28^null ^and CD28^+ ^T cells were seen on CD8^+ ^T cells from patients (data not shown). CD4^+^CD28^+^, CD8^+^CD28^+ ^and CD8^+^CD28^null ^T cells from healthy controls showed no significant expression of PRRs. CD4^+^CD28^null ^T cells from healthy controls expressed PRR to some extent but, as the prevalence of this subpopulation is low, no significant data could be acquired (data not shown).

### LPS-mediated perforin production of CD4^+^CD28^null ^T cells depends on CD14 and TLR4

**Figure 4:**
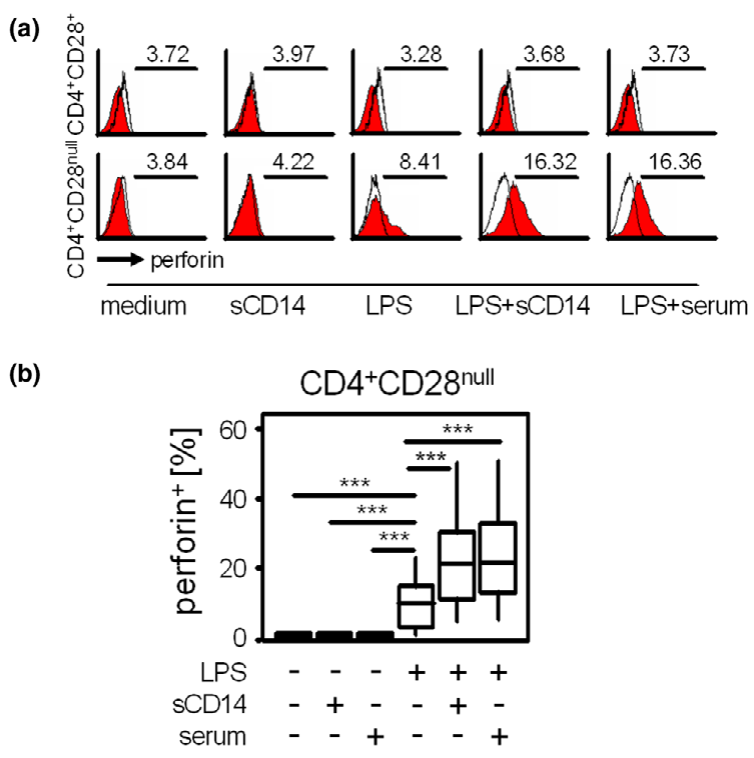
Effects of LPS, sCD14 and autologous serum on perforin production by CD4^+ ^T cells. Fresh peripheral blood mononuclear cells of patients with ankylosing spondylitis were incubated with medium (as a negative control), soluble CD14 (sCD14) or 10 μg/ml lipopolysaccharide (LPS) alone or with LPS in combination with 25 μg/ml sCD14 and 5% autologous serum for 16 hours. After staining with fluorescence-marked monoclonal antibodies directed against perforin, CD4 and CD28, cells were counted by flow cytometry. **(a) **Histograms show perforin production by CD4^+^CD28^+ ^(upper row) and CD4^+^CD28^null ^T cells (lower row) in response to medium, sCD14, LPS, LPS + sCD14 and LPS + serum as indicated (red curves). Black lines represent isotype control staining. Values indicate the mean fluorescence intensity. Gates for CD4^+^CD28^null ^and CD4^+^CD28^+ ^T cells were set as shown in Figure 3a. **(b) **Box blots show percentages of perforin-producing CD4^+^CD28^null ^T cells from seven independent experiments. Differences were tested for significance using the Wilcoxon test. ***P ≤ 0.001.

For functional testing of TLR-mediated lymphocytic stimulation, fresh PBMCs were incubated for 16 hours with LPS and the T cells were analysed for their intracellular production of perforin. As shown in Figure 4a,b, perforin was produced upon LPS stimulation by CD4^+^CD28^null ^T cells (13 ± 10.7% perforin^+ ^cells), but perforin expression was negligible in CD4^+^CD28^+ ^T cells (0.4% ± 0.3% perforin^+ ^cells, *P *= 0.009). Combining recombinant sCD14 with LPS doubled the percentage of perforin^+^CD4^+^CD28^null ^T cells (24 ± 15.3% perforin^+ ^cells) compared with LPS stimulation alone (*P *= 0.0001). A comparable additional effect was seen when cultures stimulated with LPS were supplemented with 5% autologous serum (26.9 ± 16.6% versus 13 ± 10.7% perforin^+ ^cells, *P *= 0.001) but not with FCS (data not shown). CD4^+^CD28^+ ^T cells did not produce perforin after co-incubation with LPS, even after addition of sCD14 or autologous serum (Figure 4a and data not shown).

**Figure 5:**
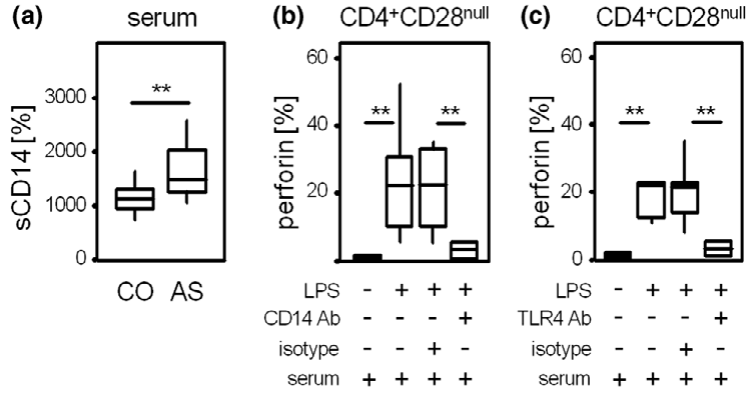
CD14 and TLR4-mediated effects. **(a) **ELISA assays were performed to analyse levels of soluble CD14 (sCD14) in sera from patients with ankylosing spondylitis (AS) (*n *= 50) and healthy controls (CO) (*n *= 23). The Mann-Whitney test was used to determine the statistical differences between the group of patients and the control group. **P < 0.01. A blocking antibody (Ab) directed against **(b) **CD14 and **(c) **TLR4 or an isotype control were added to peripheral blood mononuclear cells from patients with AS maintained in 5% autologous serum. After 1 hour, lipopolysaccharide (LPS) stimulation at a concentration of 10 μg/ml for 16 hours was started. Box blots show percentages of perforin-producing CD4^+^CD28^null ^T cells from seven independent experiments. Differences were tested for significance using the Wilcoxon test. **P < 0.01.

These findings implicated the possible occurrence of natural sCD14 in sera from AS patients. Blinded samples from patients with AS and from healthy controls were therefore analysed using enzyme-linked immunoassays. As shown in Figure 5a, the levels of sCD14 were higher in AS patients than in healthy controls (1,653.6 ± 463 pg/ml versus 1,170 ± 259 pg/ml, *P *= 0.008). To investigate whether sCD14 from autologous serum was crucial for a stronger response of CD4^+^CD28^null ^T cells to LPS, cells were incubated with a blocking antibody directed against CD14 prior to the addition of LPS. This antibody is capable of binding both sCD14 and mCD14. As expected, LPS-induced perforin production of CD4^+^CD28^null ^T cells was reversed by blocking sCD14 and mCD14 (24.0 ± 16.3% versus 3.1 ± 2.5% perforin^+ ^cells, *P *= 0.006) but not by isotype control antibody (Figure 5b).

Blocking assays with antibodies directed against TLR4 were then performed to specifically address the role of TLR4 in LPS-mediated perforin production of CD4^+^CD28^null ^T cells. As shown in Figure 5c, preincubation with anti-TLR4 antibody inhibited activation of CD4^+^CD28^null ^T cells by LPS (3.4 ± 2.3% with anti-TLR4 antibody versus 23.8 ± 14.7% perforin^+ ^cells with isotype control antibody, *P *= 0.002).

### Effects of TNF-α *in vitro *and therapeutic blockade of TNF-α *in vivo *on PRR expression of CD4^+^CD28^null ^T cells

**Figure 6:**
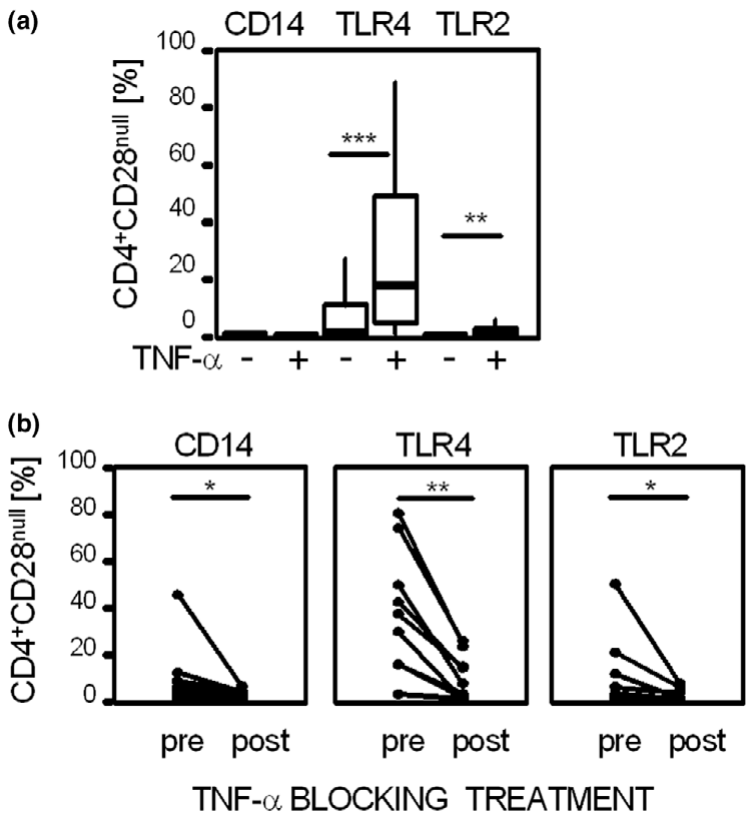
Effects of TNF-α on expression of pattern recognition receptors *in vitro *and *in vivo*. **(a) **Peripheral blood mononuclear cells were stimulated with 20 ng/ml tumour necrosis factor-α (TNF-α) for 24 hours, and CD4^+^CD28^null ^T cells were analysed for expression of CD14, TLR4 and TLR2. Box plots summarize data from seven independent experiments. Medians were compared using the Wilcoxon test. ***P < 0.001, **P < 0.01. **(b) **CD4^+^CD28^null ^T cells in patients with active ankylosing spondylitis treated with infliximab at a dosage of 3 mg/kg body weight were tested for the expression of pattern recognition receptors (PRRs) before and 3 weeks after injection (*n *= 8). The expression of CD14, TLR4 and TLR2 was detected by flow cytometry. The Wilcoxon test was used to determine differences in expression of PRRs before (pre) and under successful TNF-α blocking treatment (post). **P < 0.01, *P < 0.05.

*In vitro *assays were performed to test the effect of TNF-α on the expression of PRRs. Incubation of PBMCs with TNF-α for 24 hours increased the expression of TLR4 and TLR2, but not of CD14 on CD4^+^CD28^null ^T cells (from 9.2 ± 25.8% to 26.6 ± 27.7% for TLR4, *P *< 0.001 and from 1.1 ± 3.1% to 2.4 ± 4.1% for TLR2, *P *= 0.008; Figure 6a). Expression of CD14, TLR4 and TLR2 were neither induced on CD4^+^CD28^+ ^T cells nor on CD8^+ ^T cells after incubation with TNF-α (data not shown).

To examine the effects of TNF-α blocking treatment on the expression of PRRs on CD4^+^CD28^null ^T cells *in vivo*, peripheral CD4^+^CD28^null ^T cells from AS patients were tested before and after successful treatment with TNF-α-specific chimeric antibodies. As shown in Figure 6b, CD4^+^CD28^null ^T cells from patients during active AS disease showed higher levels of PRRs than after successful treatment with the TNF-α blocking agent. CD14 was reduced from 10.6 ± 16.6% before treatment to 2.5 ± 2% after treatment (*P *= 0.011), TLR4 was reduced from 46.9 ± 32.7% to 11.7 ± 12.5% (*P *= 0.008) and TLR2 was reduced from 11.7 ± 19.4% to 1.8 ± 2.9% (*P *= 0.012).

## Discussion

The present study shows an increased expression of PRRs on human circulating CD4^+ ^T cells lacking the CD28 co-stimulatory molecule. TLR4 can be considered an alternative signalling pathway for cytotoxic CD4^+^CD28^null ^T cells, but neither for their CD28^+ ^counterparts nor for CD8^+ ^T cells. The concomitant expression of T-cell receptor (TCR) and PRRs on the cell surface further supports the role of CD4^+^CD28^null ^T cells as an immunological link between the adaptive and the innate defence system, and is in accordance with earlier descriptions of co-existing NK receptors and TCR on these cells [25]. In all chronic immune diseases tested (AS, PsA and RA) more CD4^+^CD28^null ^T cells expressed TLR4 than TLR2, thus stressing the superior role of TLR4 over TLR2. Indeed, a significant surface expression of TLR2 on CD4^+^CD28^null ^T cells has only been found in patients with AS, but not in patients with PsA and RA (Figure 1b), which is consistent with an earlier histological study in RA that did not find TLR2 on CD3^+ ^T cells in the synovial tissue [32].

To assure a high purity of T-cell populations was a critical part in this study. T cells were therefore not only purified by MACS^® ^technology for the RT-PCR assays, but were also monocyte depleted. A high purity of CD3^+^CD4^+^CD28^null ^cells ranging from 94.2% up to 99.1% was thus obtained, as shown in Figure 2a. In a separate approach, surface expression of TLRs was studied by fluorescence-activated cell sorting analysis with gates carefully set to focus on the lymphocyte population on the forward scatter and the side scatter. An additional gate was then set on the population expressing high levels of CD4, ensuring monocytes that express lower levels of CD4 were excluded [33]. Detection of mRNA and surface expression of TLRs were therefore used as two independent techniques to ensure the presence of TLRs in the examined cell populations.

From the functional perspective, TLR4 is the key receptor for Gram-negative bacteria. In our *in vitro *model the effect of bacterial exposure on the cytotoxic function of CD4^+^CD28^null ^T cells was simulated by addition of LPS from an *E. coli *strain. TLR4 binds LPS and thus provides activating signals to the CD4^+^CD28^null ^T cells, which can be reversed with TLR4 blocking antibodies (Figure 5c). This mechanism clearly depends on CD14, which allows signal transmission [34]. CD14 in AS may either occur on cell membranes of CD4^+^CD28^null ^T cells (Figure 3b) or as a soluble molecule in the serum (Figure 5a). As the percentages of CD4^+^CD28^null ^T cells expressing mCD14 were much lower than the percentages of cells expressing TLR4, we added either recombinant CD14 or autologous sera for stimulation assays (Figure 4a,b). Indeed, addition of CD14 nearly doubled the percentage of perforin^+^CD4^+^CD28^null ^T cells upon LPS stimulation, whereas the addition of anti-CD14 antibody completely abolished the effect of LPS (Figure 5b). LPS binding protein, another TLR-related molecule, is also known to support binding of LPS to TLR4. Serum levels of LPS binding protein correlate with inflammation in RA and reactive arthritis [35], but have not so far been studied in AS. In our experiments both the addition of recombinant sCD14 and autologous serum had a comparable additional effect on LPS-induced perforin production of CD4^+^CD28^null ^T cells, which indicates serum LPS binding protein not to be indispensable in AS. Taking these facts together, activated CD4^+^CD28^null ^T cells produce perforin upon LPS-mediated activation in a CD14-dependent and TLR4-dependent manner.

As we used PBMCs for functional assays, we cannot exclude that LPS also activated antigen-presenting cells within the PBMCs. However, direct LPS-mediated effects on TLR4^+ ^T cells appear more relevant: TLR^- ^T cells were not activated in the presence of LPS, and the percentage of perforin^+^CD4^+^CD28^null ^T cells correlated well with the prevalence of TLR4-expressing CD4^+^CD28^null ^T cells (data not shown). Antigen-presenting cells would not need addition of CD14 for activation by LPS anyway, as CD14 is widely expressed on antigen-presenting cells.

Direct TLR-mediated activation of human T cells has been previously shown for activated CD8^+ ^T cells and CD4^+^CD45RO^+ ^memory T cells from healthy individuals with high surface levels of TLR2, but not TLR4 [17]. Although a number of CD4^+^CD28^+ ^T cells express the memory marker CD45RO (data not shown), we did not detect TLR2 on these cells. A possible explanation for the discrepancy with our results may be that the mAbs used recognize different epitopes or variants of TLRs. We showed that expression of mRNA for TLR2 was present in both CD4^+^CD28^+ ^and CD4^+^CD28^null ^T cells to a varying extent (Figure 2b). In contrast, we found TLR4 exclusively in CD4^+^CD28^null ^T cells on both the mRNA and the protein level. The activation of TLR4 on CD4^+^CD28^null ^T cells was independent of TCR-mediated stimulation for perforin production, and TLR4 signalling did not lead to an additive effect on concomitant cross-linking of TCR (data not shown). The high affinity of TLR4 to LPS without the obligatory need of the TCR signal may therefore have an influence on the susceptibility of CD4^+^CD28^null ^T cells from AS patients to Gram-negative bacterial components.

As TNF-α directly influences CD28 gene transcription and may facilitate the emergence of CD4^+^CD28^null ^T cells in chronic inflammatory syndromes [36], we also studied the effects of TNF-α on PRRs *in vitro*. In line with its effects on monocytic TLRs on the mRNA level [37], TNF-α also resulted in an increased protein expression of TLR4 and TLR2 on CD4^+^CD28^null ^T cells (Figure 6a). Accordingly, expression of TLRs on CD4^+^CD28^null ^T cells from patients with active AS disease before treatment (Figure 6b) were higher than those of unselected AS patients (examined for Figure 3b). The expression of CD14, TLR4 and TLR2 was then reduced on fresh CD4^+^CD28^null ^T cells from AS patients treated with TNF-α blocking agents, further indicating the important role of TNF-α for the upregulation of surface expression of these PRRs also on CD4^+^CD28^null ^T cells (Figure 6b).

## Conclusion

The finding of PRRs on cytotoxic CD4^+^CD28^null ^T cells of patients with AS, PsA or RA represents a new pathophysiological link between the innate and the adaptive immune system. *In vitro *activation of CD4^+^CD28^null ^T cells by LPS is mediated by TLR4 and depends on CD14. Additional work has to be carried out to explain the downstream mechanisms of action and the clinical implications of these findings.

## Abbreviations

AS = ankylosing spondylitis; ELISA = enzyme-linked immunosorbent assay; FCS = foetal calf serum; IFN-γ = interferon gamma; IL = interleukin; LPS = lipopolysaccharide; mAb = monoclonal antibody; mCD14 = membrane-bound CD14; NF = nuclear factor; NK = natural killer; PBMC = peripheral blood mononuclear cell; PBS = phosphate-buffered saline; PCR = polymerase chain reaction; PRR = pattern recognition receptor; PsA = psoriatic arthritis; RA = rheumatoid arthritis; RT = reverse transcriptase; sCD14 = soluble CD14; TCR = T-cell receptor; TLR = Toll-like receptor; TNF-α = tumour necrosis factor alpha.

## Competing interests

The 'Verein zur Förderung der Hämatologie, Onkologie und Immunologie' (Innsbruck, Austria) which sponsors the laboratory, had been supported to a minor extent by Aesca, Austria. The authors declare that they have no competing interests.

## Authors' contributions

BR, C Dejaco, C Duftner and CG carried out the cell culture work, WK carried out the ELISAs. CG also helped to coordinate the study. SCV and MK performed RT-PCR. BR, C Dejaco, C Duftner and MS designed the study, performed the statistical analysis and drafted the manuscript. BGL critically provided important discussion on the data. All authors read and approved the final manuscript.

## Figures and Tables

**Table 1:** Patients' characteristics

	Controls (*n *= 64)	Ankylosing spondylitis (*n *= 65)	Psoriatic arthritis (*n *= 25)	Rheumatoid arthritis (*n *= 72)
Age (years)	49.2 ± 12.4	43.1 ± 10.9	52.2 ± 12	57.9 ± 11
Gender (% female)	50.8	28.4	45.8	75.6
Rheumatoid factor positivity (%)	0.0	n.d.	n.d.	77.8
CCP-Ab positivity (%)	0.0	n.d.	n.d.	80.5
HLA-B27 positivity (%)	n.d.	78.6	31.3	n.d.
Axial involvement (% positive)	0.0	100.0	48.0	0.0
Erythrocyte sedimentation rate >15 mm/hour (% positive)	0.0	62.7	88.2	86.2
C-reactive protein >0.7 μg/dl (% positive)	0.0	26.1	41.7	46.8

CCP-Ab, antibodies directed against cyclic citrullinated peptides; n.d., not determined.
